# Research on maize canopy center recognition based on nonsignificant color difference segmentation

**DOI:** 10.1371/journal.pone.0202366

**Published:** 2018-09-27

**Authors:** Xiushan Wang, Hehu Zhang, Ying Chen

**Affiliations:** 1 Department of Electrical Engineering, College of Mechanical & Electrical Engineering of Henan Agricultural University, Zhengzhou, Henan, China; 2 College of Humanities & Law of Henan Agricultural University, Zhengzhou, Henan,China; College of Agricultural Sciences, UNITED STATES

## Abstract

Weed control is a substantial challenge in field management. A better weed control method at an earlier growth stage is important for increasing yields. As a promising weed control technique, intelligent weeding based on machine vision can avoid the harm of chemical weeding. For machine vision, it is critical to extract and segment crops from their background. However, there is still no optimal solution for object tracking with occlusion under a similar color background. In this study, it was found that the gray distribution of a maize canopy follows the gradient law. Therefore, the recognition method based on the HLS-SVM (HLS color space and Support Vector Machine) and on the grayscale gradient was developed. First, the HLS color space was used to detect the maize canopy. Second, the SVM method was used to segment the central region of the maize canopy. Finally, the maize canopy center was identified according to the gradient law. The results showed that the average segmentation time was 0.49 s, the average segmentation quality was 87.25%, and the standard deviation of the segmentation was 3.57%. The average recognition rate of the center position was 93.33%. This study provided a machine vision method for intelligent weeding agricultural equipment as well as a theoretical reference for further agricultural machine vision research.

## Introduction

Maize is one of the three largest food crops in the world, and approximately 1/3 of the people in the world use maize as its primary food source [1]. In addition, maize is also one of the main feedstuff for developing animal husbandry. Reasonable field management can ensure maize yields [2]. However, in the early stage of maize growth, serious weed damages always make maize plant growth extremely slow. Weed control is an important field management method to improve maize yields. Weed control methods are mainly divided into manual weeding, chemical weeding, mechanical weeding and automatic intelligent weeding. Due to the aging of the population and the rise of labor prices, time-consuming and tedious manual weeding has been gradually replaced by simple and effective chemical weeding. However, the chemical herbicides lead to environmental pollution as well as reduced maize nutrition and increased weed drug resistance. In recent years, mechanical weeding has been used instead of chemical weeding. However, mechanical weeding without a perceptual system easily damages the roots and stems of crops [3]. With the rapid development of machine vision, intelligent agricultural equipment is developing rapidly [4]. Intelligent agricultural equipment with machine vision is more promising than traditional agricultural equipment in fruit harvesting, weed control, and navigation in the field. Some examples are provided as follows. Machine vision is used to recognize seedlings’ position in transplanting [5], the thinning machine is used to recognize excessive sugar beet plants based on machine vision [6], the intelligent weed machine is used to identify the weeds based on machine vision [7, 8], the tomato cluster picking robot is used to identify tomatoes based on machine vision [9], and machine vision was used to locate the positions of green peppers [10]. Although machine vision is widely used in agriculture, due to weather and complex backgrounds, the recognition rate and positioning accuracy are not high. There is still no good solution for object tracking with occlusion under similar color backgrounds. Furthermore, image segmentation is important and is perhaps the most difficult task that has a direct effect on the recognition results [11]. In agriculture image processing, some image segmentations are often adopted, such as color index threshold-based and learning-based segmentation. The segmentation method based on the color index is mainly divided into two categories: the green index segmentation method based on the different weights of color components and the comprehensive method based on different green index methods. For example, the color index of vegetation extraction (CIVE) was proposed by Kataoka in 2003 [12]. The standardized green—red difference index (NGRDI) was proposed by hunt in 2005 [13]. The vegetation index (VEG) for separating plant (grains and weeds) pixels from soil pixels was proposed by Hague in 2006 [14]. The segmentation methods based on learning are mainly divided into two categories. One category is the unsupervised learning method. For example, the fuzzy clustering unsupervised learning method was proposed by Meyer in 2004 [15]. The other category is supervised learning. For example, the supervised mean shift algorithm based on back propagation neural networks was proposed by Zheng in 2009 [16]. To solve the problem of illumination, the learning method based on a decision tree was proposed by Guo in 2013 [17].

All the above methods and applications are mainly focused on plant segmentation and recognition, and constantly improving the segmentation effect is the key to solve all the recognition, classification and location problems. It is easy to use machine vision to locate the target under significant color differences, but target recognition under similar color backgrounds does not have a mature, effective and universal solution. Therefore, under similar color background characteristics, the real-time maize canopy center position recognition needs to be solved urgently. This research work developed the maize canopy center recognition method based on the HLS-SVM (HLS color space and Support Vector Machine)and gray gradient law and achieved the accurate identification of maize center positions. The method is divided into three stages. First, an RGB image was converted to an HLS image, and the green vegetations were segmented from the background using HLS color segmentation [18]. Then, the SVM classifier was used to identify the blue maize canopy’s central region, namely, the regions of interest (ROI), and the residual vegetation region, namely, the non-regions of interest (N-ROI) [19–21]. Since some weed regions have a similar color to the ROI, the weeds cannot be completely removed by SVM segmentation. Fortunately, the weeds and the ROI were isolated from each other after SVM segmentation, and the ROI occupied the most area in the image. Therefore, the remaining weeds were filtered out using the area filtration method. As weeds and maize canopy cover each other, small weeds were still connected around the ROI after filtering. To eliminate the weeds, the morphological processing method was used to obtain the smooth ROI [22]. Finally, the maize canopy center was further identified from the ROI. Before identifying the canopy center, the gray distribution of the ROI was analyzed. It was found that all the grayscale images, from the canopy center to the canopy periphery, followed the increasing gradient law, which is the unique attribute of the chlorophyll distribution of the maize canopy. This feature can be used to segment the different central hierarchies of the canopy, which provides a favorable condition for more accurate canopy center identification. The statistics showed that the gray level of the maize canopy center was concentrated with 0~30 gray values. Therefore, the threshold of the gray level was set to 30, and the maize canopy center was identified by threshold segmentation.

## Materials and methods

### Materials

#### Experimental field and maize plant

The research was carried out in the experimental base of the National Maize Cooperative Innovation Center in China. Maize plants were planted according to the agronomic requirements to facilitate the mechanization and identification of the intelligent equipment. The distance of each line was 60 cm, and the distance between two strains was 40 cm. The video was collected from the spring sowing maize canopy. The average height of the maize plant was 40–60 cm, and the speed of the video tracking car was 1.2 m/s.

#### Machine visual experimental platform parameters

Video acquisition peripherals: A video orbiter, a camera rocker arm 15 meters long, and 10 straight cars.

Camera parameters: The Q-PRI high speed camera is a portable high-speed camera made by AOS (AOS Technologies AG) in Switzerland. A 3 million pixel resolution is suitable for the image acquisition of fast moving objects. The frame rate of this resolution is up to 500 frames per second.

Video processing hardware: CPU: Intel(R)core™ i5-6500 @3.3 GHz. Processor architecture: x86_64. The core number of the processor is 4, the number of threads per core is 1, and the cache size is 6144 KB. The memory capacity is 4G, and the data hard disk’s capacity is 1 T.

Video processing software: operating system: ubuntu 16.04. Video image processing language: C++ and Python.

#### Recognition method of the maize canopy center position

This part mainly studies the recognition method for the maize canopy center position. The four main steps are as follows.

1. **Global segmentation based on HLS mask:** The RGB image is converted to an HLS image, and the color detection method is used to segment the green vegetation from the background.
2. **Local segmentation based on Linear SVM:** The SVM classifier is used to extract the ROI in the green vegetation.
3. **Canopy center recognition based on the grayscale gradient law:** According to the grayscale increasing phenomenon from the canopy center to the canopy periphery, the canopy center position is identified from the ROI.

#### Global segmentation based on HLS mask

**Fig 1:**
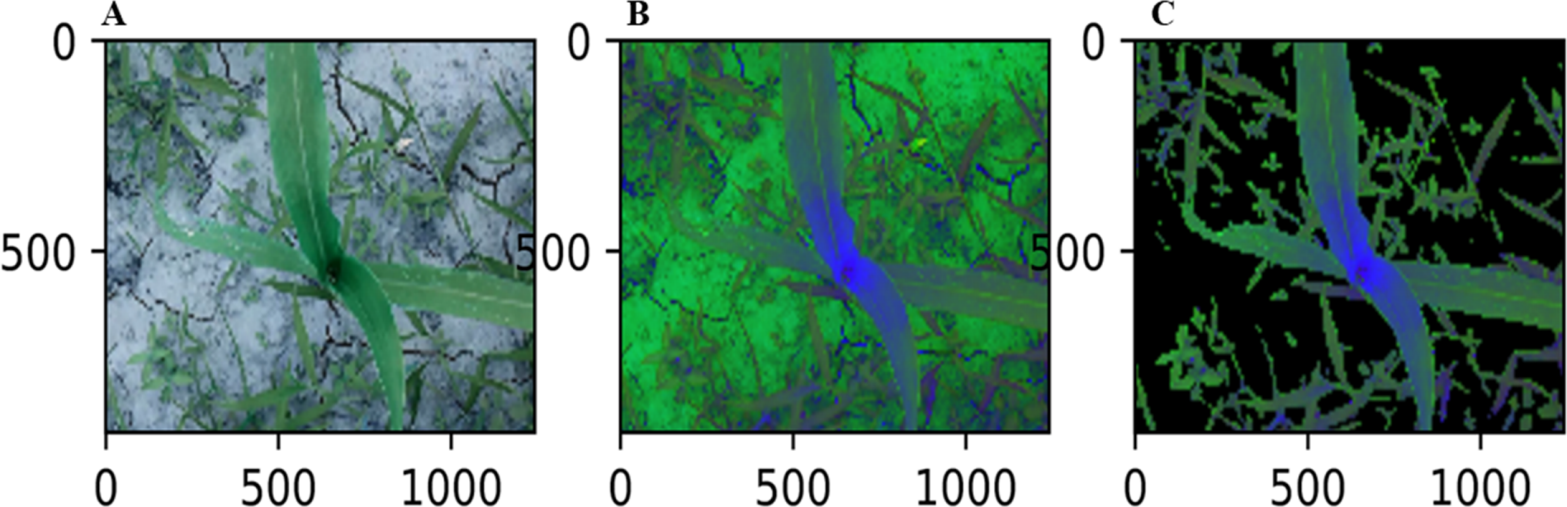
Global segmentation based on HLS mask. (A)RGB image.(B)HLS image.(C)Mask image.

To identify the maize canopy center, the background first need to be filtered out. In previous studies, there are some methods that were used, such as the Standard Deviation Index, Excess Green Index, Excess Red Index, Color Index of Vegetation Extraction, and Combined Ex-G and Ex-GR and CIVE and VEG indices, as well as the Hue Index. This article uses the HLS space mask to filter the background. First, the image is converted from the RGB color space to the HLS space (Fig 1A and 1B), and then it is filtered with the HLS-mask (Fig 1C). The HLS thresholds is given by the expression

$$Mask_{hls}\left(x,y\right)=\{\begin{array}{ll} 1 & if:35<H<99,0<L<255,0<S<255\\ 0 & else \end{array}$$ (1)

where x and y are pixel coordinates in the image, H is the hue of the image, L is the brightness of the image, and R is the saturation component of the image.

The color distribution is mainly concentrated on two regions. The central region (the ROI) given by expression (2) is dark blue, and the surrounding region (the N-ROI) is green. The canopy center is located in the ROI, and the ROI will be segmented again.

$$\mathrm{ROI}=I(x,y)*Mask_{hls}(x,y)$$ (2)

where I is the HLS image, and ROI is the region(s) of interest in the HLS image, namely, the central region of the maize canopy.

#### Local segmentation based on linear SVM

**Fig 2:**
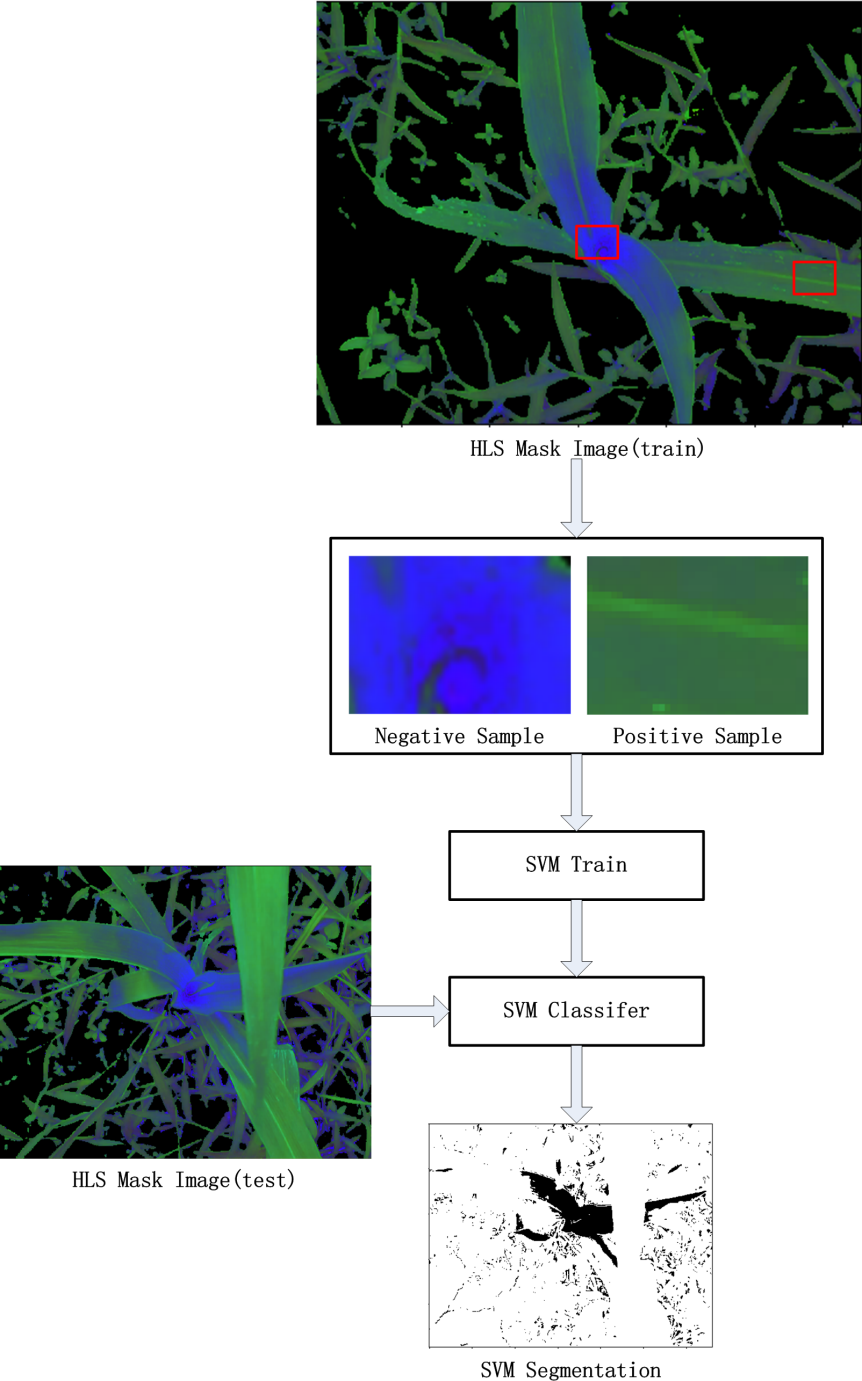
Local segmentation based on the Linear SVM. Select the candidate region from the ROI as the positive training sample, and select the candidate region from the N-ROI as the negative training sample. Then, fit the SVM model, and finally the SVM classifier is used to segment the HLS image.

To segment the ROI, the SVM classifier is used to identify the ROI and N-ROI. The SVM is a machine learning method, which is a model based on the statistical learning theory and the structural risk minimization proposed by Vapnik [23, 24]. Because of the excellent learning ability of the SVM and the relatively low over fitting rate, the SVM model has good classification performance [25]. The local segmentation based on the SVM is described as follows (Fig 2).

The local segmentation based on the Linear SVM includes four steps: training, parameter optimization, testing, and post processing. The next section is the detailed implementation of the SVM segmentation model.

**(1) SVM kernel function selection and parameter optimization**

**Fig 3:**
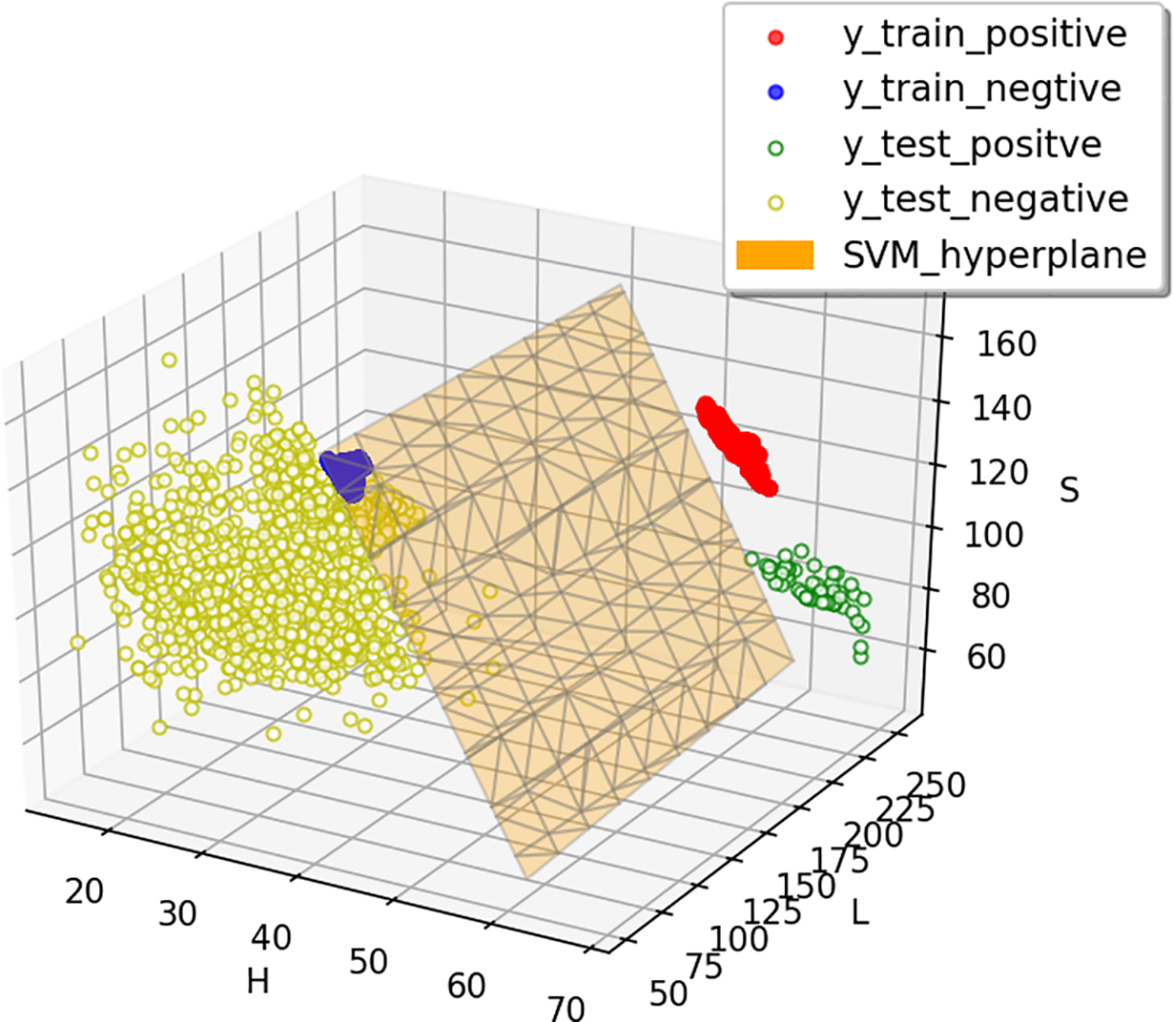
Linear SVM hyper-plane and green vegetation pixel distribution in the HLS color space.

To judge whether the ROI and the N-ROI in the HLS image are linearly separable, it is necessary to understand the distribution state of the two region’s pixels in the color space. The pixels in the training and the testing images are mapped to the HLS color space, respectively (Fig 3). The red and the blue solid points represent the positive and negative training samples, respectively, which are all distributed in two different regions. The green and the yellow hollow points represent the ROI and the N-ROI of the canopy, respectively. It can be seen that the two kinds of pixels be separated by the Linear SVM classification hyper-plane. Therefore, by using the Linear SVM kernel, the ROI and N-ROI can be separated. The SVM classification function is shown in formula (2).

$$Map_{svm}\left(\vec{x}\right)=\mathrm{sgn}\left(a_{i}y_{i}\left({\vec{x}}_{i}\cdot \vec{x}\right)+b\right)$$ (3)

where the vector $\vec{x}=\mathrm{Vector}(\mathrm{ROI})$, the vectors ${\vec{x}}_{i}\left(1,2,\ldots ,N\right)$, and N is the number of samples.

The predicted results are stored in the label vector *V*_*label*_(*n*), as shown in formula (4).

$$V_{label}\left(n\right)=\left(Classifer_{svm}(Map_{svm},V_{test}\left(n\right)\right)$$ (4)

Convert *V*_*label*_ to a matrix named *Mask*_*svm*_, as shown in formula (5).

$$Mask_{svm}=Matrix(V_{label}(n))=\left[\begin{array}{cccc} +1 & +1 & -1 & \ldots \\ -1 & +1 & -1 & \ldots \\ -1 & -1 & -1 & \ldots \\ \ldots & \ldots & \ldots & \ldots \end{array}\right]$$ (5)

After selecting the SVM kernel function, the cross validation is performed to get the best parameters, including the penalty factor *C* and the slack variable *g*. In addition, select the best sample size according to the SVM learning curve. The training sets and the 4-fold cross validation method are used to build the Linear SVM model.

**Fig 4:**
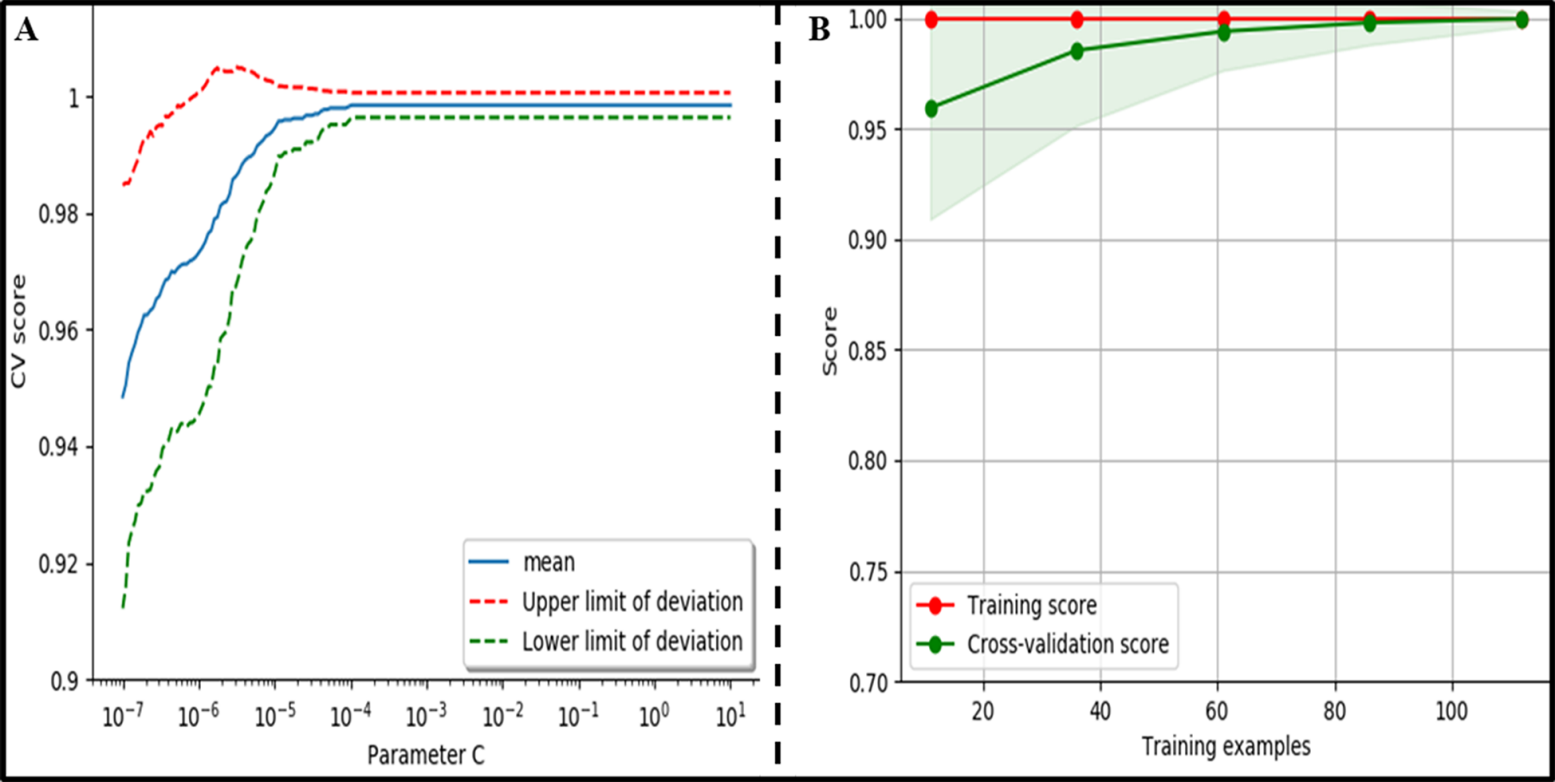
SVM parameter. (A) C cross validation curves.(B) Learning curves(Linear Kernel,g = 0.001).

By the above cross validation, it is found that the variable *g* does not significantly affect the performance of the Linear SVM, and it is set to auto. Therefore, the penalty factor *C* is used as the main parameter that is analyzed. The red and the green dashed lines respectively represent the upper and lower variances, and the blue solid line represents the average cross validation curve (Fig 4A). It can be seen that when *C* is small, the average variance of the score is very large, and the score increases with the increase of *C*. After *C* ≥ 0.1, the variance gradually becomes small and stable. Thus, *C* = 0.1 is used as the best parameter.

The characteristics of the support vector make the classifier obtain very good classification results with only a few training samples, and there is no serious over-fitting phenomenon. The red curve is the training score, the green curve is the cross validation score, and the light green area is the variance (Fig 4B). With the sample number increasing, the score rate increases, and the variance decreases. When the sample size is 100, the score of cross validation is the largest, and the variance is the smallest at the same time. Therefore, the sample size of 100 is selected.

**(2) Post processing of the SVM segmentation image**

It can be seen from the HSL mask image that both the ROI and some of the surrounding weeds appear blue. After the SVM segmentation, the blue weeds cannot be completely removed, and those need to be removed by the post processing.

**Fig 5:**
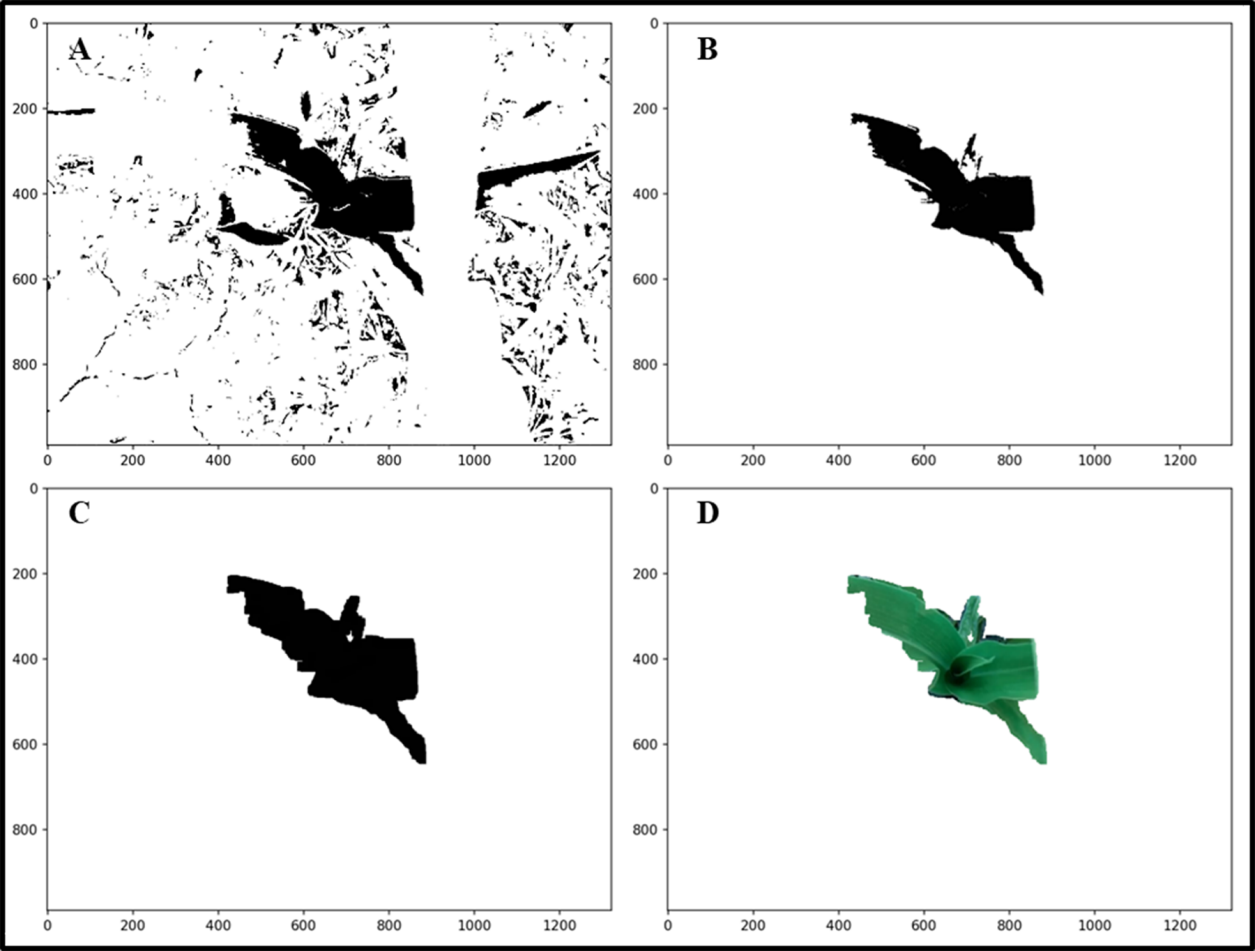
The post processing of the SVM segmentation. (A)SVM segmentation.(B) Area filter.(C)Erosion.(D)ROI.

After the SVM segmentation, the ROI are separated from most of the weeds, and the ROI occupied most of the image. As a result, the rest of the weeds can be filtered out using the area filtering method (Fig 5A and 5B). Then, the morphological methods are used to conduct erosion operations on the ROI to eliminate the residual boundary areas (Fig 5C). The image is eroded with the structure element $K=\left[\begin{array}{ccc} 1 & 1 & 1\\ 1 & 1 & 1\\ 1 & 1 & 1 \end{array}\right]$ and the convolution is defined as follows:

$$\left(Mask_{svm}*K\right)\left(x,y\right)=\min\left\{Mask_{svm}\left(x+x^{\prime },y+y^{\prime }\right)-K\left(x^{\prime },y^{\prime }\right)|\left(x^{\prime },y^{\prime }\right)\in D_{s}\right\}$$ (6)

where *D*_*s*_ is the domain of S, *x*,*y* is the pixel position of *Mask*_*svm*_, and *x*′,*y*′ is the pixel position of the Kernel. After the area filtering and morphological processing, the residual weeds are filtered out and the ROI mask is retained (Fig 5D).

#### Recognition of the canopy center based on grayscale gradient

**Fig 6:**
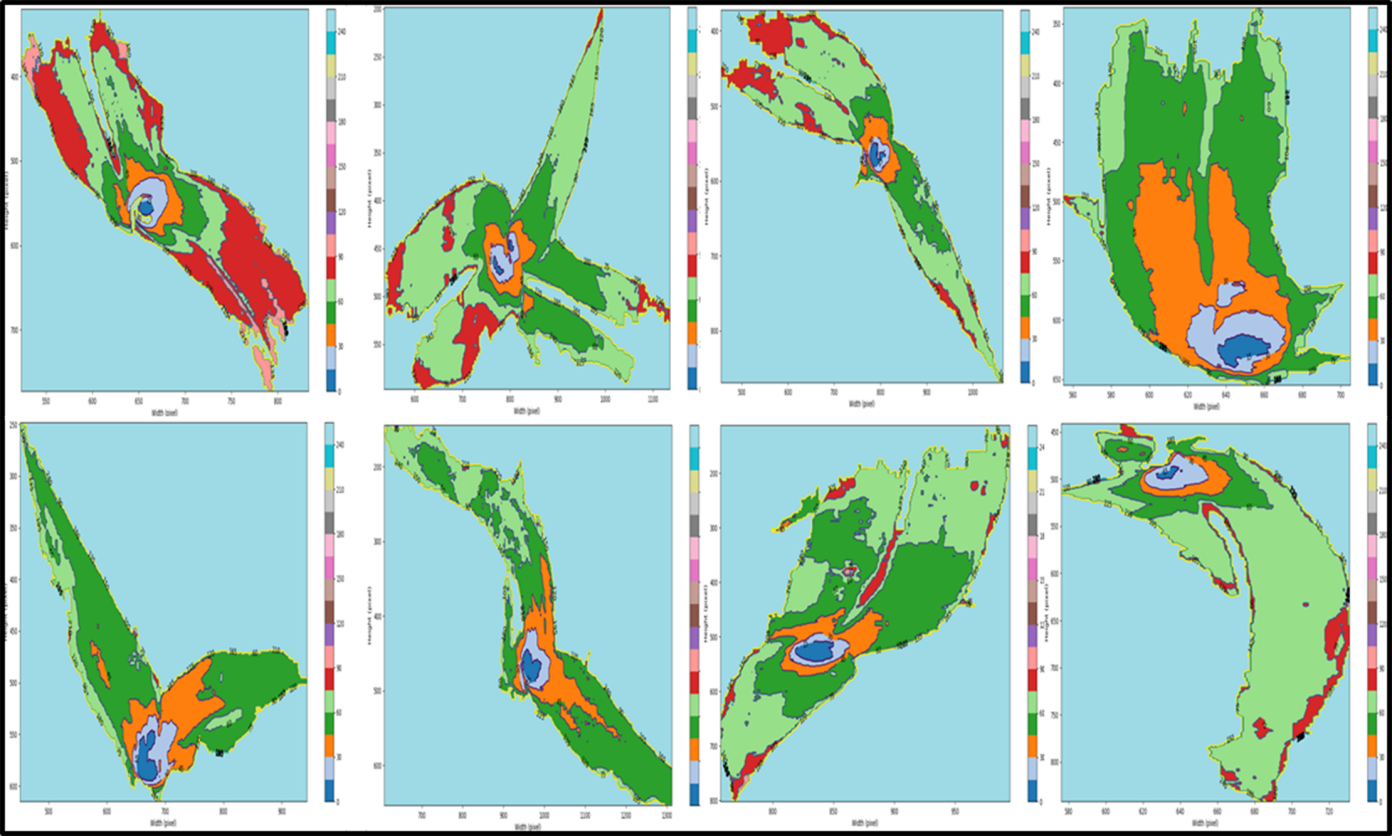
Gray gradient distribution in the ROI. The gray level of 0–255 is divided into 17 parts, and the tab 20-color set is used to coloring the different gradation regions of the grayscale image in the ROI. The isohypse from low to high is [15,30,45,60,75,90,105,120,135,150,165,180,195,210,225,240,255].

The ultimate aim is to acquire the maize canopy’s center position. The results revealed that the ROI gray value follows the gradient law, which is the essential feature of the maize leaf Chlorophyll distribution (Fig 6). This feature can be used to segment the ROI. Furthermore, it provides a favorable condition for more accurate center identification.

Although the increasing gray level is not linearly related to its radius, it is positively correlated with it. After statistical analysis, it is found that the gray level in the ROI is mainly concentrated on the gray range of 0~30, and therefore the maize canopy center can be identified accurately by the gray threshold segmentation.

## Results

### Results of local segmentation based on the Linear SVM

**Fig 7:**
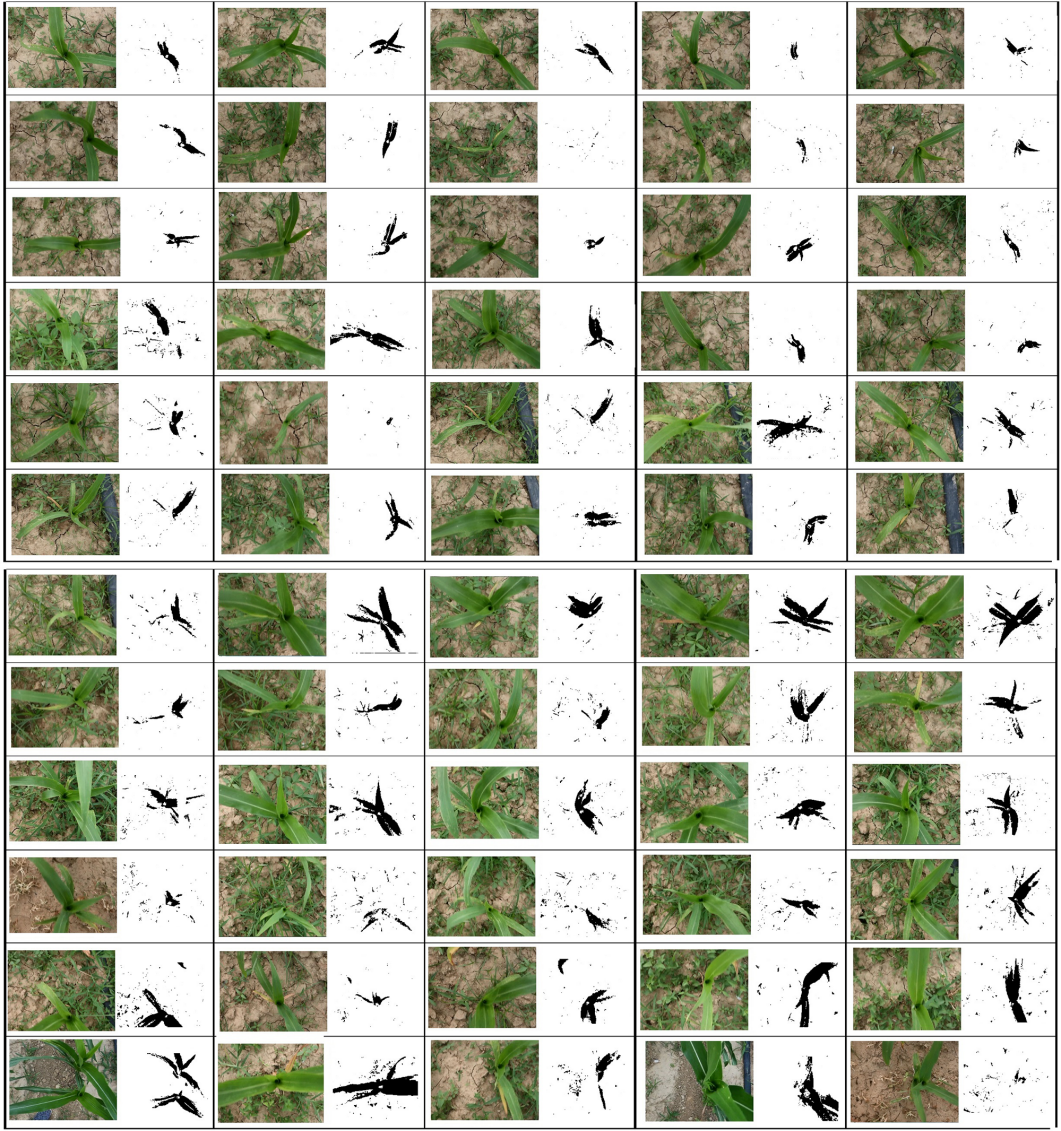
Linear SVM classifier segmentation results for HLS Mask images.

We display the 60 frame segmentation results in which the 57 ROI are extracted, but there are still 3 frame images that show a poor segmentation effect (Fig 7). When the size of the canopy is similar to the size of the weeds, the color distribution of the ROI in the HLS space is very close to the weeds and the surrounding leaves of the maize canopy, and the SVM classifier mistakenly identifies the ROI as a weed.

### Results of canopy center recognition based on the grayscale gradient law

**Fig 8:**
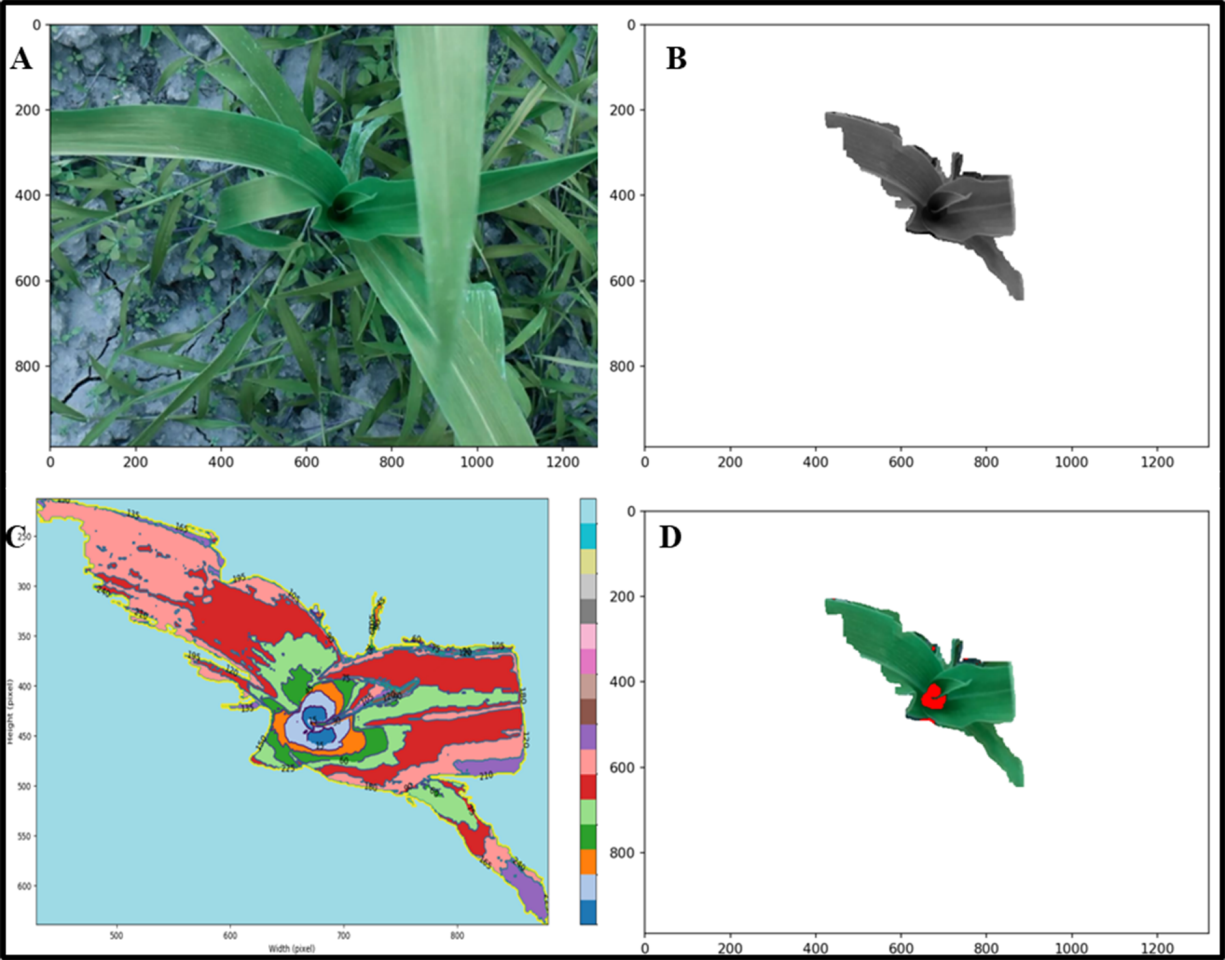
The results of the canopy center recognition based on the gray gradient distribution. (A)Canopy.(B)Gray ROI.(C)Gray gradient ROI.(D)ROI center.

Previously, the HSL color and Linear SVM segmentation methods have been used to obtain the ROI. The ROI's RGB image needs to be converted to the gray image (Fig 8A and 8B), and then the gray gradient distribution in the ROI is analyzed (Fig 8C). Therefore, we can use the gray threshold method to set the gray threshold T< 30 (Fig 8D).

### Statistics of the maize canopy center recognition

**Fig 9:**
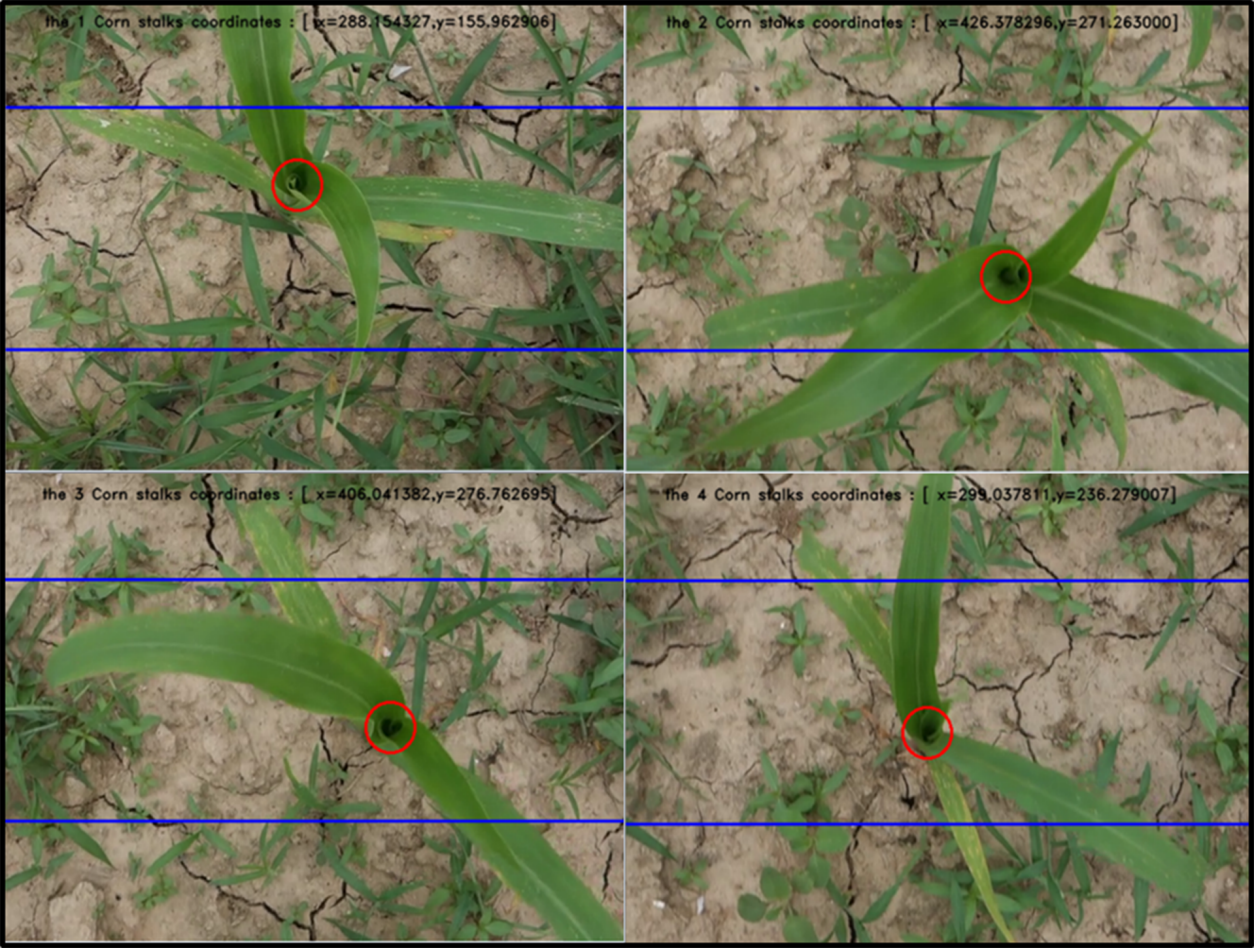
Statistics of the recognition rate of the maize canopy center. The x and y values: the canopy center location; the area between the two blue lines: the recognition area; and the red circle: the center of the recognition.

The maize canopy centers are identified by this method, and the canopy centers of each group are counted. The identification results of the 4 frame images are displayed (Fig 9).

The canopy centers of the 1641 images in 12 groups are identified, and the test results showed that the average recognition rate of the canopy layer was 93.33% (Table 1).

**Table 1:** Statistics of the maize canopy center recognition rate.

Group number	1	2	3	4	5	6	7	8	9	10	11	12
**Quantity**	122	145	118	129	123	150	152	148	145	143	142	124
**Recognition rate: %**	91	93	99	91	92	93	99	93	93	99	78	99

## Discussions

### Performance of local segmentation based on the Linear SVM

**Fig 10:**
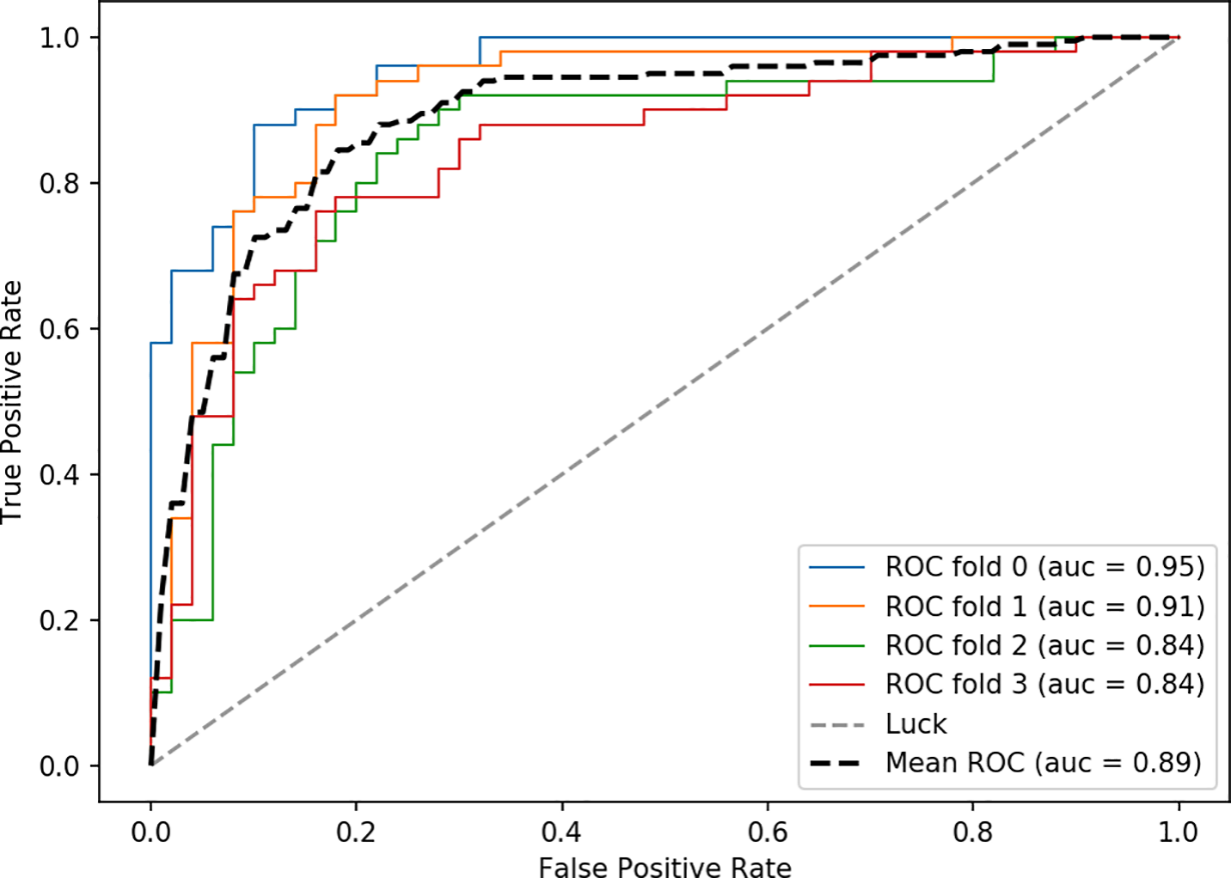
Receiver operating characteristic curve for the SVM classifier.

The ROC (receiver operating characteristic) curves and AUC (area under the curve) are often used to evaluate a binary classifier. AUC is a very common evaluation index in machine learning and classification models. The larger the AUC is, the better the correct classification performs. The training set was used for four cross tests to calculate the AUC and the average AUC of each cross validation (Fig 10). The red, green, blue, and orange curves are the four cross validation ROC curves, the rough black dotted line is the average ROC, and the light colored dotted line is the reference. It can be seen from the graph that the AUC of the mean ROC is 0.89, and the Linear SVM has a lower error rate when classifying HLS images.

### Comparison of local segmentation performance based on the different methods

The accuracy of the segmentation methods is evaluated by the following performance measurements [26].

$$Q_{seg}=\frac{\sum_{i=0}^{i=m}\sum_{j=0}^{j=n}\left(A\left(v\right)i,j\cap B\left(v\right)i,j\right)}{\sum_{i=0}^{i=m}\sum_{j=0}^{j=n}\left(A\left(v\right)i,j\cup B\left(v\right)i,j\right)}$$ (7)

where *A* is the set of the vegetation pixels (*v* = 0), *B* is a reference set of manually segmented vegetation pixels (*v* = 0), *m*,*n* are the respective image row and column sizes, *i*,*j* are the pixel coordinate indices of the images, and Q_*seg*_ is the consistency of both the vegetation part and background part. The mean of the segmentation quality factor and the standard deviation of the evaluation image set can be derived using Q_*seg*_. Table 2 is a comparison of the ROI segmentation methods for each image with the size of 1242x931 and the resolution of 96 dpi under the same conditions.

**Table 2:** Comparison of the ROI segmentation methods.

Model	Mean of segmentation quality (μ%)	Standard Deviation (σ%)	Average computation time(s)
**Ex-G**	75.68	7.21	0.352
**COM1**	78.15	6.34	0.361
**Linear SVM**	82.14	5.33	0.521
**HLS-SVM**	87.25	3.57	0.490

The average time consumption based on the HLS-SVM method is 0.49 s, which is slower than the color index-based Ex-G and COM1 methods, but a difference was not obvious among them. In addition, the time consumption of the HLS-SVM is shorter than that of the single SVM method because the HLS method filters a large number of the background pixels and reduces the numbers of segmentation objects. From the mean of segmentation quality and the standard deviation, the segmentation effect of the HLS-SVM is better than the others, which results from the improvement of the computation complexity.

### Comparison of local segmentation performance under different weathers and times

**Fig 11:**
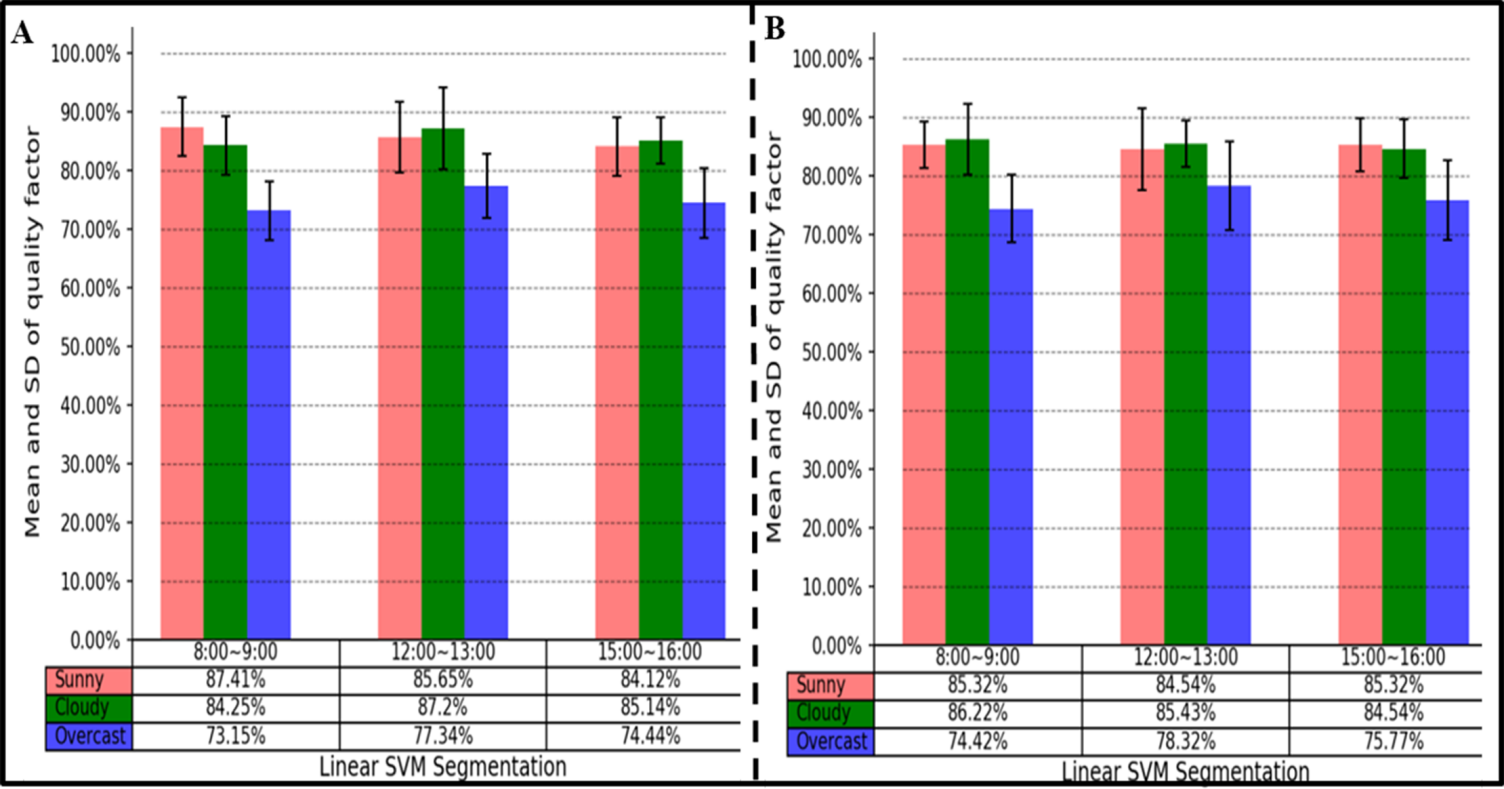
Mean and SD of quality factor. (A) A samples from Zhengzhou.(B) B samples from Nanyang. Red indicates sunny days, Green indicates cloudy, and Blue indicates overcast. Each histogram is divided into three time periods. The table below the histogram records the average value of the quality of the segmentation under different circumstances.

The ROI segmentation performance under different weather conditions and times was analyzed. The validation set with 600 samples was collected from two contrasting experimental fields under the following conditions: the times were 8:00, 12:00, and 16:00, and the weather included Sunday cloudy overcast. The SD of the quality with little change indicated that the HLS-SVM method had robustness under different weather conditions and times (Fig 11A and 11B). In addition, it was found that the method had a better segmentation effect during clear mornings or during the midday. This finding shows that the soft sunlight has a good effect on the HLS-SVM method.

During distinguishing similar colors, the machine learning method with high segmentation precision has a strong sensitive to the training data source while the color index method with low segmentation precision can quickly filter and eliminate a large number of unrelated backgrounds. Combination of the two methods above can achieve complementary advantages. The new method enables the weed robot to identify the maize canopy more accurately under similar green background while maintaining real-time performance. And then the gradient method was used to quantitatively reveal the distribution of the gray level of the canopy, which makes the weeding robot more accurately perceive plant location and reduce the risk of damage to plants.

## Conclusions

In this paper, a novel recognition method for maize canopy centers based on machine vision technology was developed. The method combined the HLS-SVM theory with the gray gradient distribution rule and achieved the accurate position identification of the maize canopy under similar color backgrounds using multi-level segmentation. The recognition effect was better than the single identification method and is suitable for real-time identification using intelligent weeding. The research provided a complete machine vision system for intelligent weeding agricultural equipment as well as a theoretical reference for further agricultural machine vision research.
